# A preclinical model of cutaneous melanoma based on reconstructed human epidermis

**DOI:** 10.1038/s41598-022-19307-0

**Published:** 2022-09-29

**Authors:** Anna Leikeim, Maximiliane Wußmann, Freia F. Schmidt, Nuno G. B. Neto, Franziska Benz, Kendra Tiltmann, Corinna Junger, Michael G. Monaghan, Bastian Schilling, Florian K. Groeber-Becker

**Affiliations:** 1 Department Tissue Engineering & Regenerative Medicine (TERM), University Hospital Würzburg, Röntgenring 11, 97070 Würzburg, Germany; 2 Translational Center for Regenerative Therapies TLZ-RT, Fraunhofer-Institute for Silicate Research ISC, Würzburg, Germany; 3 Department of Mechanical, Manufacturing and Biomedical Engineering, Trinity College Dublin, Dublin, Ireland; 4 Trinity Centre for Biomedical Engineering, Trinity College Dublin, Dublin, Ireland; 5 Advanced Materials and Bioengineering Research Centre (AMBER), Royal College of Surgeons in Ireland and Trinity College Dublin, Dublin, Ireland; 6 CÚRAM, Centre for Research in Medical Devices, National University of Ireland, Newcastle Road, Galway, H91 W2TY Ireland; 7 Department of Dermatology, University Hospital Würzburg, Würzburg, Germany

**Keywords:** Melanoma, Oncology

## Abstract

Malignant melanoma is among the tumor entities with the highest increase of incidence worldwide. To elucidate melanoma progression and develop new effective therapies, rodent models are commonly used. While these do not adequately reflect human physiology, two-dimensional cell cultures lack crucial elements of the tumor microenvironment. To address this shortcoming, we have developed a melanoma skin equivalent based on an open-source epidermal model. Melanoma cell lines with different driver mutations were incorporated into these models forming distinguishable tumor aggregates within a stratified epidermis. Although barrier properties of the skin equivalents were not affected by incorporation of melanoma cells, their presence resulted in a higher metabolic activity indicated by an increased glucose consumption. Furthermore, we re-isolated single cells from the models to characterize the proliferation state within the respective model. The applicability of our model for tumor therapeutics was demonstrated by treatment with a commonly used v-raf murine sarcoma viral oncogene homolog B (BRAF) inhibitor vemurafenib. This selective BRAF inhibitor successfully reduced tumor growth in the models harboring BRAF-mutated melanoma cells. Hence, our model is a promising tool to investigate melanoma development and as a preclinical model for drug discovery.

## Introduction

Despite accounting for less than 5% of all skin cancers, melanoma is responsible for up to 90% of skin cancer-related deaths worldwide^1,2^. The high mortality rate of melanoma patients is predominantly due to the propensity of early metastases as well as to the rapid evolvement of therapeutic resistance^3,4^. The highly aggressive nature of melanoma emphasizes the need for early diagnosis, as well as the development of new therapeutic strategies.

Melanoma develops from pigment-producing melanocytes, localized in the basal layer of the epidermis, by malignant transformation (melanomagenesis). Genetic dispositions and environmental influences are both causal for malignant transformation, with ultraviolet radiation emerging as the most important exogenous risk factor^5^.

To investigate melanomagenesis and the effect of anticancer drugs, early research relied on adherent cultures of tumor cells. However, since the cells here are grown in monoculture on an artificial plastic surface, conventional two-dimensional (2D) cell culture represents only a reductionist approach, lacking not only the respective microenvironment, but exhibiting altered proliferation rates and aberrant responds to drugs. Thus, neglecting the dynamic interactions of adjacent cells, drug screening approaches are prone to misinterpretation^6^.

Between simplistic 2D cultures of melanoma cell lines and complex animal models, there are several approaches to study melanoma in vitro. Three-dimensional (3D) models represent a good compromise between the lack of microenvironment in 2D cell cultures and the complexity of animal models^6,7^. The first attempts for a 3D arrangement of the tumor cells are spheroids, which can be generated from tumor cells alone or combined with other tumor-associated cells e.g., fibroblasts^8^ or endothelial cells^9^. In comparison to 2D cell culture, spheroids enable cell–cell-contacts as well as the formation of a heterogeneous tumor mass caused by an oxygen and nutrient gradient^10–14^. However, spheroids reflect solely the tumor and not the surrounding tissue, in which the tumor develops. Hence, tumor-tissue-interaction is not sufficiently reflected, limiting the predictability in drug testing. Furthermore, topically administered drugs cannot be tested in spheroids. Skin models mimic the anatomy of human skin and thus could serve as an elegant model to study the formation of melanoma and its treatment or cellular and molecular crosstalk between the tumor and the surrounding skin cells. This approach has been used in different studies, mainly by integrating melanoma cell lines in epidermal-dermal models^15–26^. These full-thickness skin models are used in numerous research projects but have not been implemented in regulatory accepted guidelines yet due to a more challenging standardization. However, to be applicable in preclinical studies the models need to allow up-scaling and reproducibility. This emphasizes the need for the development of less complex 3D in vitro test systems, such as Reconstructed Human Epidermis (RHE). RHE with integrated melanomas could bridge the gap between the simplicity of conventional 2D cell culture or spheroids and the complexity of full thickness skin equivalents and animal models. These test systems provide adequate human tissue representation while controlling the number of variables within the system. In 2020, 416 therapies against malignant melanoma were in preclinical phases and required a significant number of animal models for efficacy and safety testing^27^. Thus, the usage of RHE with integrated melanomas will support the 3R principle first described by Russel and Burch^28^ by reducing the number of animal experiments within the preclinical phases of the development of anti-melanoma therapies.

In this study, we present an accessible 3D melanoma model that harbours not only the tumor itself but also partly the respective microenvironment, in which the melanomagenesis takes place. We have established a methodology that allows the tumor to mature within a physiologically differentiated epidermis. This model is based on the open-source reconstructed human epidermis (OS-REp) model, ensuring that a broad scientific community can benefit from this research by adopting the freely published protocol for different scientific questions. To generate the model, different melanoma cell lines were introduced to a keratinocyte suspension before cell seeding. Thereby defined tumors with different driver mutations can be generated that are applicable for in vitro drug discovery and efficacy testing. Furthermore, analytical methods for the evaluation of therapeutic effects were developed specifically for the requirements of the 3D in vitro tissue models and assessed by vemurafenib treatment.

## Materials and methods

### Generation of melanoma models

Primary human epidermal keratinocytes were isolated from foreskin biopsies from juvenile donors according to a previously published protocol^29^. This study was approved by the ethics committee of the University of Würzburg (approval number 182/10 and 280/18) and conducted according to the Helsinki Declaration. Samples were obtained only after the informed consent of the legal guardian(s).

Melanoma cell lines SK-MEL-28, MM96L, A11, D08 and MM127 were kindly provided by Prof. Dr. Nick Hayward, Queensland Institute for Medical Research (Brisbane, Australia). Melanoma cell line BLM was kindly offered by Prof. Dr. Marc Schmidt, Department of Dermatology at the University Hospital Würzburg (Würzburg, Germany). Furthermore, MUG-Mel2 were kindly provided by Assoz. Prof. Priv.-Doz. Mag. Dr. Beate Rinner, Medical University Graz (Graz, Austria). MeWo, A375 and Malme3M were purchased from American Type Culture Collection. Keratinocytes were cultured in E1 medium (EpiLife® medium supplemented with 1% human keratinocytes growth supplements (HKGS) and 1% penicillin/streptomycin (all Thermo Fisher, USA)). Melanoma cell lines were cultured in RPMI medium (Thermo Fisher) containing 10% fetal calf serum (FCS; Bio & Sell, Germany) and 1% penicillin/streptomycin (Thermo Fisher).

Melanoma models are based on a previously published protocol^29^. Briefly, keratinocytes in passage 3 were mixed with different ratios of melanoma cells in E2 medium (E1 medium supplemented with 1.44 mM CaCl_2_). Subsequently, a defined number of melanoma cells were seeded with 3 × 10^5^ keratinocytes on a polycarbonate membrane (pore size 0.4 µm) of 12-well inserts (Merck Millipore, Darmstadt, Germany). After 24 h, medium was changed to E3 medium (E2 medium containing 10 ng/ml Keratinocyte Growth Factor (Thermo Fisher) and 73 μg/ml ascorbin-2-phosphat) and models were lifted to the air–liquid-interphase. Media was changed three times per week for a period of 20 days.

### Assessment of glucose consumption

Glucose consumption was analyzed photometrically in cell culture supernatants using the Cedex Bio Analyzer (Roche, Germany). Consumption was determined at day 20 of culture and was calculated by subtracting the measured glucose concentrations from the value for fresh medium (6.44 mM).

Detection of local glucose uptake was performed with 2-(N-(7-Nitrobenz-2-oxa-1,3-diazol-4-yl)Amino)-2-Desoxyglucose (2-NBDG). The models were incubated in 100 µM 2-NBDG for 60 min at 37 °C. For qualitative analysis of glucose uptake, fluorescence images were taken employing a Biorevo BZ-9000 microscope (Keyence Corporation, Japan).

### Two-photon fluorescence lifetime imaging microscopy (2-P FLIM)

Two-Photon Fluorescence Lifetime Imaging Microscopy (2-P FLIM) was performed on a custom multiphoton system. A titanium:sapphire laser (Chameleon, Coherent®) was used for multiphoton excitation. A water-immersion 25 × objective (Olympus, 1.05NA) was utilized on an upright (Olympus BX61WI) laser scanning microscope. Two-photon excitation of nicotinamide adenine dinucleotide (NAD(P)H) and flavin adenine dinucleotide (FAD^+^) fluorescence was performed with 760 nm and 800 nm excitation wavelength, respectively. A 455/90 nm bandpass filter was used to isolate NAD(P)H fluorescence signal and a 502/47 nm bandpass filter for FAD^+^ fluorescence emission. 512 × 512 pixel images were acquired with a pixel dwell time of 3.81 μs and 30-s collection time. A PicoHarp 300 TCSPC system operating in the time-tagged mode coupled with a PMA hybrid detector (PicoQuanT GmbH, Germany) was used for fluorescence decay measurements yielding 256 time bins per pixel. For each field of view, both NAD(P)H and FAD^+^ images were captured and at least 3 images for each model were acquired. Afterwards, regions of interest (ROI) were selected and the NAD(P)H fluorescence decay and FAD^+^ fluorescence intensity were analysed.

For the NAD(P)H fluorescence decay analysis, an overall decay curve was generated by the contribution of all pixels in the ROI area. Afterwards, it was fitted with a double exponential decay curve [Eq. (1)]:

1 $$I\left(t\right)={\alpha }_{1}{e}^{-\frac{t}{{\tau }_{1}}}+{\alpha }_{2}{e}^{-\frac{t}{{\tau }_{2}}}+c$$

*I(t)* corresponds to the fluorescence intensity measured at time *t* after laser excitation; *α*_*1*_ and *α*_*2*_ represent the fraction of the overall signal comprise of a short and long component lifetime component, respectively. *τ*_*1*_ and *τ*_*2*_ are the short and long lifetime components. *C* corresponds to background light.

X^2^ value is calculated to evaluate the goodness of multiexponential fit to the raw fluorescence decay data. In this study the lowest χ^2^ values were considered.

For NAD(P)H, a two-component fit was used to differentiate between the free (*τ*_*1*_) and protein-bound (*τ*_*2*_) NAD(P)H. The average lifetime (*τ*_ave_) of NAD(P)H for each pixel is calculated by a weighted average of both free and bound lifetime contributions [Eq. (2)]:

2 $${\tau }_{ave} =\frac{\left({\alpha }_{1}\times {\tau }_{1}\right)+({\alpha }_{2}\times {\tau }_{2})}{({\alpha }_{1}+{\alpha }_{2})}$$

For FAD^+^ only its fluorescence intensity was taken into account. With the addition of NAD(P)H fluorescence intensity it is possible to calculate the optical redox ratio (ORR) using Eq. (3):

3 $$ORR=\frac{NAD\left(P\right)H}{NAD\left(P\right)H+{FAD}^{+}}$$

### Measurement of skin barrier function

Transepidermal water loss (TEWL) was measured at day 20 of culture to analyze the integrity of the skin barrier using a device made by Courage and Khazaka electronic GmbH (Köln, Germany) according to the manufacturer’s specifications. Measurements started 10 min after placing the device on top of the wells to minimalize air turbulences. Water loss was measured for 10 min at 37 °C^30^.

Additionally, skin barrier function was analyzed via impedance spectroscopy as described previously^31^. Briefly, on day 20 of culture, models were placed between a working and a counter electrode of a custom-made measuring system. Space between the stainless-steel electrodes was filled with EpiLife® medium supplemented with 1% penicillin/streptomycin and 1.44 mM CaCl_2_. A spectrum with 40 logarithmic distributed measuring frequencies between 1 Hz and 100 kHz was measured using the impedance spectrometer LCR HiTESTER 3522–50 (HIOKI E.E. Corporation, Japan).

### Histological assessment

For histological assessment, tissue samples were fixed in 4% paraformaldehyde, embedded in paraffin and 4 µm histological cross sections were prepared. For a morphological overview of the models, hydrated cross sections were stained with hematoxylin and eosin (HE). Immunohistochemical stainings were performed using the SuperVision 2 HRP Kit (DCS Innovative Diagnostik-Systeme, Germany) according to the manufacturer’s instructions. The following antibodies were used: recombinant monoclonal rabbit anti-Ki67 (Cat# ab16667, 1:100, Abcam, United Kingdom), recombinant monoclonal rabbit anti-S100β (Cat# ab52642, 1:100, Abcam), monoclonal mouse anti-melanosome/HMB-45 (Cat# M0634, 1:50, Agilent Dako, USA), monoclonal mouse anti-Melan-A (Cat# M7196, 1:50, Agilent Dako), monoclonal mouse anti-microphthalmia-associated transcription factor (MiTF, Cat# ab80651, 1:500, Abcam)). Counterstaining was performed with hematoxylin. For immunohistological analysis of re-isolated cells, cells in suspension were sedimented on glass slides by centrifugation to generate cytospots. Cells on dried slides were fixed using 4% paraformaldehyde and blocked using 5% BSA in phosphate-buffered saline (Sigma-Aldrich) containing 0.2% Triton™ X-100 (Sigma-Aldrich) for 20 min at room temperature. Cytospots were incubated with primary antibodies against Ki67, Melan-A or cytokeratin 14 (polyclonal rabbit anti-KRT14, Cat# HPA023040, 1:1000, Sigma-Aldrich) overnight at 4 °C. Slides were washed and subsequently incubated with fluorophore-conjugated secondary antibody (polyclonal donkey anti-mouse Alexa Fluor 647 (Cat# A31571) or polyclonal donkey anti-rabbit Alexa Fluor 647 (Cat# A31573), respectively, both 1:400, Thermo Fisher) for 1 h at room temperature. After repeated washing, slides were covered with Fluoromount-DAPI (Thermo Fisher).

### Assessment of metabolic activity

Metabolic activity was analyzed via 3-(4,5-dimethyldiazol-2-yl)-2,5-diphenyltetrazolium bromide (MTT) assays at day 20. Therefore, equivalents were incubated at 37 °C for 3 h in 1 mg/ml MTT, (Sigma-Aldrich). After removal of remaining MTT solution, the formazan salt was extracted from the tissues using 2 ml 2-propanol and quantified via absorbance measurement at 570 nm employing a spectrophotometer (Infinite 200 M; Tecan). Values were normalized to the untreated replicates of each group.

### Treatment of melanoma models with the BRAF inhibitor vemurafenib

To ensure a standardized treatment, vemurafenib (PLX4032, Selleckchem, Germany) was dissolved in E3 medium to defined concentrations and applied for 72 h. Compound was refreshed during media change after 48 h.

### Re-isolation of keratinocytes and melanoma cells

For re-isolation of single cells, models were placed in dispase at a concentration of 2 U/ml (corresponds to 33.34 nkat/ml) for 5 min. The epidermis was detached from the membrane using tweezers and placed in trypsin to isolate keratinocytes. After 10 min, enzymatic reaction was stopped with 10% FCS. To isolate melanoma cells, the detached epidermis was placed in accutase® for 25 min. After enzymatic treatment, cells were separated mechanically by rapid resuspending.

### Cell cycle analysis

The different cell cycle phases were distinguished by determination of deoxyribonucleic acid (DNA) content using flow cytometry. DNA was stained with propidium iodide (Sigma Aldrich). Since the fluorescence intensity is proportional to the DNA content, this signal can be measured to determine the cell cycle phase. After staining, the cells were washed and fluorescence intensity was assessed using a FACSCalibur™ (BD Biosciences, USA).

### Statistical analysis

All data are depicted as mean values (n ≥ 3) with standard deviation (SD). Statistical analysis was performed using GraphPad Prism 8 software (GraphPad Software Inc., USA). Gaussian distribution was tested with the Shapiro–Wilk test. Normally distributed data were analyzed using an unpaired t test or one-way analysis of variance (ANOVA) followed by Dunnett’s multiple comparisons test. If data were not normally distributed, a Mann–Whitney test or Kruskal–Wallis test with Dunn’s multiple comparisons test was performed. Mean values were compared to the respective control (OS-REp or 0 µM vemurafenib). A p-value of ≤ 0.05 was considered as statistically significant and is indicated by a star throughout the manuscript. All experiments were conducted in three independent test runs with a minimum of three technical replicates.

## Results

### Melanoma models can be generated using different melanoma cell lines

Ten melanoma cell lines with different driver mutations were used to generate melanoma epidermal models (mOS-REp) based on the OS-REp protocol (Table 1).

**Table 1:** Tested melanoma cell lines.

Cell line	Driver mutation	Origin	Growth in 2D	Growth in 3D
MM96L	BRAF^V600E^	Human, lymph node metastasis	+	++
SK-MEL-28	BRAF^V600E^	Human, skin	++	++
MM127	NRAS^G13R^	Human, subcutaneous metastasis	++	−
A11	BRAF^WT^ NRAS^WT^	Human, unknown	++	+
D08	NRAS^Q61K^	Human, melanoma	+	−
MUG-Mel2	NRAS^Q61R^	Human, cutaneous metastasis	++	++
MeWo	CDKN2A	Human, lymph node metastasis	++	++
A375	BRAF^V600E^	Human, skin	++	−
Malme3M	BRAF^V600E^	Human, lymph lung metastasis	+	−
BLM	NRAS	Human, obtained by injection of parent cell line (BRO) in nude mice and selection of cells from lung metastasis	+	−

Ten different melanoma cell lines were used to generate melanoma skin models. Differences were observed in their growth properties in 2D and 3D. Although all cells could be cultured in 2D, only 5 cell lines formed tumors in the 3D models. Growth in 2D: ++ normal cell growth; + slower growth. Growth in 3D: ++ ratio melanoma cells to keratinocytes 1:100; + ratio melanoma cells to keratinocytes higher than 1:100; – not detectable in the 3D models.

**Figure 1:**
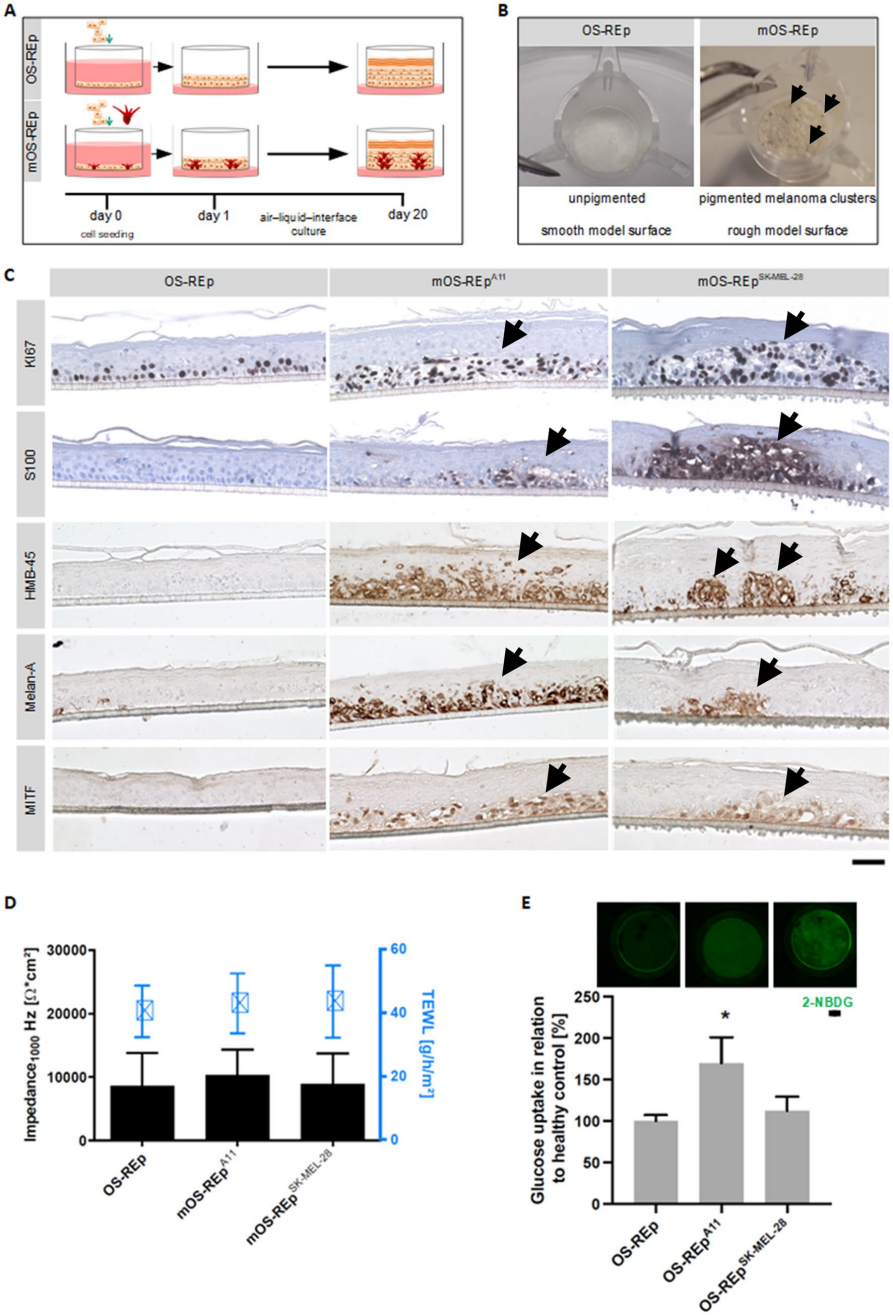
Generation and characterization of melanoma skin models. (**A**) Schematic of the generation process of the open-source epidermis (OS-REp) and melanoma models (mOS-REp). Keratinocytes alone or keratinocytes together with melanoma cells were seeded on a porous membrane on day 0. On day 1, the models were set to the air–liquid-interface and cultured until day 20. (**B**) Macroscopic images of OS-REp and mOS-REp. Formed micro-tumors of A11 appear as dark patches in the model and are marked with arrowheads. (**C**) Immunohistological staining. Melanoma models generated from the melanoma cell lines A11 (mOS-REp^A11^), SK-MEL-28 (mOS-REp^SK-MEL-28^) and healthy epidermal models (OS-REp) were stained for the proliferation marker Ki67 and the melanoma-associated markers S100, HMB-45, Melan-A and MITF. Positively stained melanoma nests are indicated by arrowheads. Scale bar 50 µm. (**D**) Analysis of barrier properties. The transepithelial electrical resistance (TEER_1000 Hz_) and the transepidermal water loss (TEWL) were determined at day 20 of culture. Incorporation of melanoma cells did not impair the barrier of the models. (**E**) Differences in glucose uptake of OS-REp and mOS-REp. Glucose consumption of the models was quantified at day 20 by measuring remaining glucose in the medium and subtracting the value from the initial glucose concentration of the medium (6.44 mM). Both mOS-REp showed an increased glucose uptake but only for mOS-REp^A11^ the increase was significant. For visualization of local glucose uptake, models were incubated with fluorescence-labelled glucose (2-NBDG) for 60 min (upper row), revealing a more intense signal for mOS-REp. Scale bar 200 µm.

To build up mOS-REp, melanoma cells were seeded with keratinocytes in a predefined ratio (Fig. 1A), ranging between 1:10 and 1:100. The optimal ratio of melanoma cells and keratinocytes was determined individually for each cell line to enable the formation of tumor nests physiologically originating from the basal layer of a well stratified epidermis. In case the integration of the respective cell line was successful, melanoma cell nests could be visualized macroscopically within the tissue equivalents after 20 days of culture at the air liquid interface (Fig. 1B). Moreover, in histological cross-sections melanoma nests were detectable by HE-staining and by immunohistochemical staining of the melanoma-associated markers MiTF, Melan-A, S100 and HMB-45 (Fig. 1C). Five out of ten melanoma cell lines formed detectable tumor nests within the OS-REp (Table 1, Fig. S1). Generation of mOS-REp with MM127, D08, A375, Malme3M and BLM cells was not successful, since no melanoma cells were detectable in the tissue models on day 20. As SK-MEL-28 and A11 represent a BRAF-mutated and a BRAF-wildtype melanoma cell line and achieved a similar histopathology compared to melanoma in vivo, further model construction focused on these two cell lines.

Both melanoma cell lines formed clusters, varying in size and shape that were distributed stochastically across the entire model (Fig. 1C). These melanoma cell clusters were primarily located in the *stratum basale.* However, depending on their size and expansion, the clusters extended into the suprabasal epidermis layers. In comparison to healthy tissue, the tumor clusters had a fissured and loose appearance, which is due to a low proportion of cell–cell contacts. Ki67 staining of mOS-REp showed a strong proliferative activity in melanoma cell clusters, but also occasionally in suprabasal areas, whereas the occurrence of Ki67-positive cells in OS-REp was restricted to the basal regions. Both mOS-REp were positive for the melanoma markers S100, HMB-45, Melan-A and MITF, while OS-REp was unambiguously negative. On closer examination, MiTF, Melan-A, S100 and HMB45 staining revealed a cell line-specific distribution of tumor cells within the tissue models. While SK-MEL-28 cells formed individual, strongly delimited tumor nests that were separated from one another by large areas of physiological epidermis, A11 cell clusters extended over large parts of the model. In A11 models, the major tumor cell quantities appeared to spread laterally, whereas SK-MEL-28 clusters seemed to migrate upwards in the direction of the *stratum corneum*.

In order to investigate whether the integration of melanoma cells impaired the barrier properties of the in vitro generated epidermis equivalents, the transepithelial electrical resistance at 1000 Hz (TEER_1000Hz_) and the TEWL were measured (Fig. 1D). Neither impedance spectroscopy nor the TEWL analysis provided evidence for the impairment of the models’ barrier properties. Without melanoma cells, OS-REp showed TEER_1000Hz_ values of 8605 Ω*cm^2^, while mOS-REp with melanoma cells reached TEER_1000Hz_ values of 8880 Ω*cm^2^ (mOS-REp^SK-MEL-28^) or up to 10,304 Ω*cm^2^ (mOS-REp^A11^). OS-REp achieved a mean TEWL value of 40.5 g/h/m^2^ whereas mOS-REp reached TEWL values of 42.9 g/h/m^2^ (mOS-REp^A11^) and 43.5 g/h/m^2^ (mOS-REp^SK-MEL-28^).

Tumor cells rely primarily on glycolysis as a main source of energy needed for cellular processes in contrast to differentiated healthy cells which rely on oxidative phosphorylation (OxPhos) as their main metabolic pathway^32^. This metabolic feature is known as the “Warburg effect”^33^. Therefore, the metabolic distinction between tumor and healthy cells in our human epidermal melanoma models was evaluated in order to provide clinically translatable results. Glucose consumption was analyzed to investigate the metabolic properties of the in vitro epidermis equivalents (Fig. 1E). Fluorescence-labeled 2-NBDG was used to visualize the spatial glucose uptake within the three-dimensional tissue equivalents by fluorescent microscopy. An increased fluorescence intensity and thus glucose uptake could be observed exclusively in melanoma models. Microscopic images revealed that glucose uptake in mOS-REp^A11^ was distributed equally over the model, while mOS-REp^SK-MEL-28^ showed heterogeneous glucose uptake in individual metabolically active areas distributed across the entire model. Global glucose consumption analyses performed from the cell culture supernatant showed a slight increase for mOS-REp^SK-MEL-28^ and a strong significant increase in mOS-REp^A11^ compared to OS-REp.

### 2-P FLIM shows distinct metabolic profiles between healthy and melanoma cells

**Figure 2:**
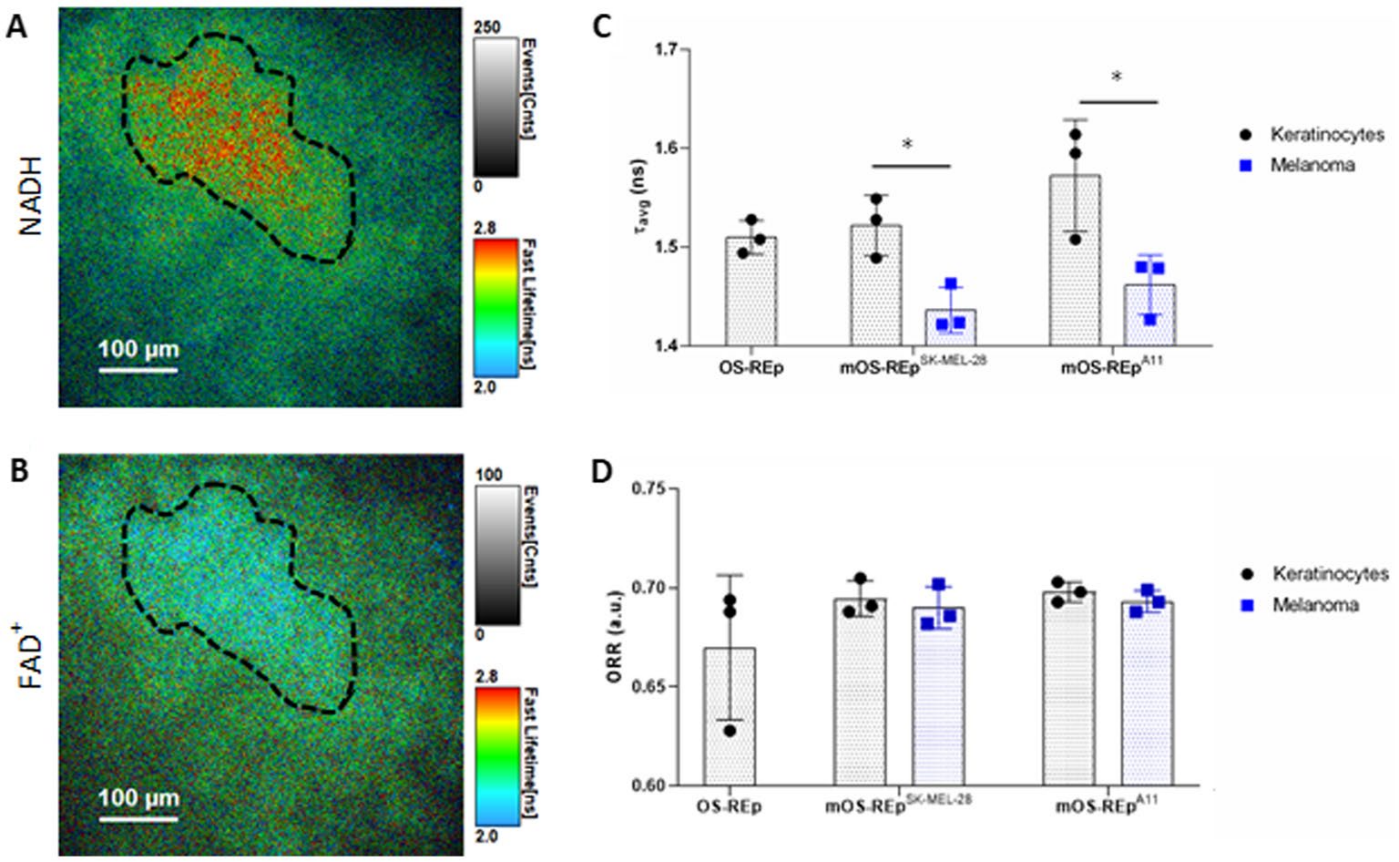
2P-FLIM of NAD(P)H and FAD^+^ analysis. (**A**, **B**) Representative NAD(P)H and FAD^+^ images of mOS-REp^A11^ with keratinocytes area highlighted (black dashes). (**C**, **D**) Calculated average fluorescence lifetimes (τ_avg_) of NAD(P)H and optical redox ratio (ORR) for OS-REp, mOS-REp^SK-MEL-28^, mOS-REp^A11^. A reduction of τ_avg_ is related with an increase in glycolysis while a reduction of ORR is associated with higher metabolic activity.

2-P FLIM is a non-invasive metabolic imaging technique which probes cellular metabolism in real-time^34–36^. Among many things, it measures the fluorescence decay and intensity of two metabolic co-factors: NAD(P)H and FAD^+^. In our results we calculated the average fluorescence lifetime (τ_avg_) of NAD(P)H and the optical redox ratio (ORR)^37,38^. To achieve this, we acquired both NAD(P)H and FAD^+^ images on the same field of view and selected two ROI distinguishing between keratinocytes and melanoma cells (Fig. 2A,B).

We detected a statistically significant decrease in τ_avg_ derived from melanoma cells when compared to keratinocytes (in both, mOS-REp^SK-MEL-28^ and mOS-REp^A11^, Fig. 2C). mOS-REp^SK-MEL-28^: 1.436 ± 0.019 ns vs 1.522 ± 0.025 ns; mOS-REp^A11^:1.462 ± 0.025 ns vs1.572 ± 0.046. This decrease in τ_avg_ results from an increase of free-NADH fraction, a consequence of a greater dependence of the melanoma cells on glycolysis^34,35^.

When calculating the ORR, a similar trend was found (Fig. 2D). The mOS-REp^SK-MEL-28^ model keratinocytes have 0.694 ± 0.007 and a slight decrease was observed when compared with its melanoma cells at 0.69 ± 0.009. For the mOS-REp^A11^ model, a small decrease was observed when comparing keratinocytes (0.698 ± 0.004) and its melanoma cells with 0.693 ± 0.004. ORR differences are associated with shifts in NAD(P)H and FAD + intracellular concentrations. This information can provide information regarding glutaminolysis and it has been shown to be correlated with tumor metastatic potential^39,40^.

### Cells show differences in cell cycle when comparing 2D and 3D culture

**Figure 3:**
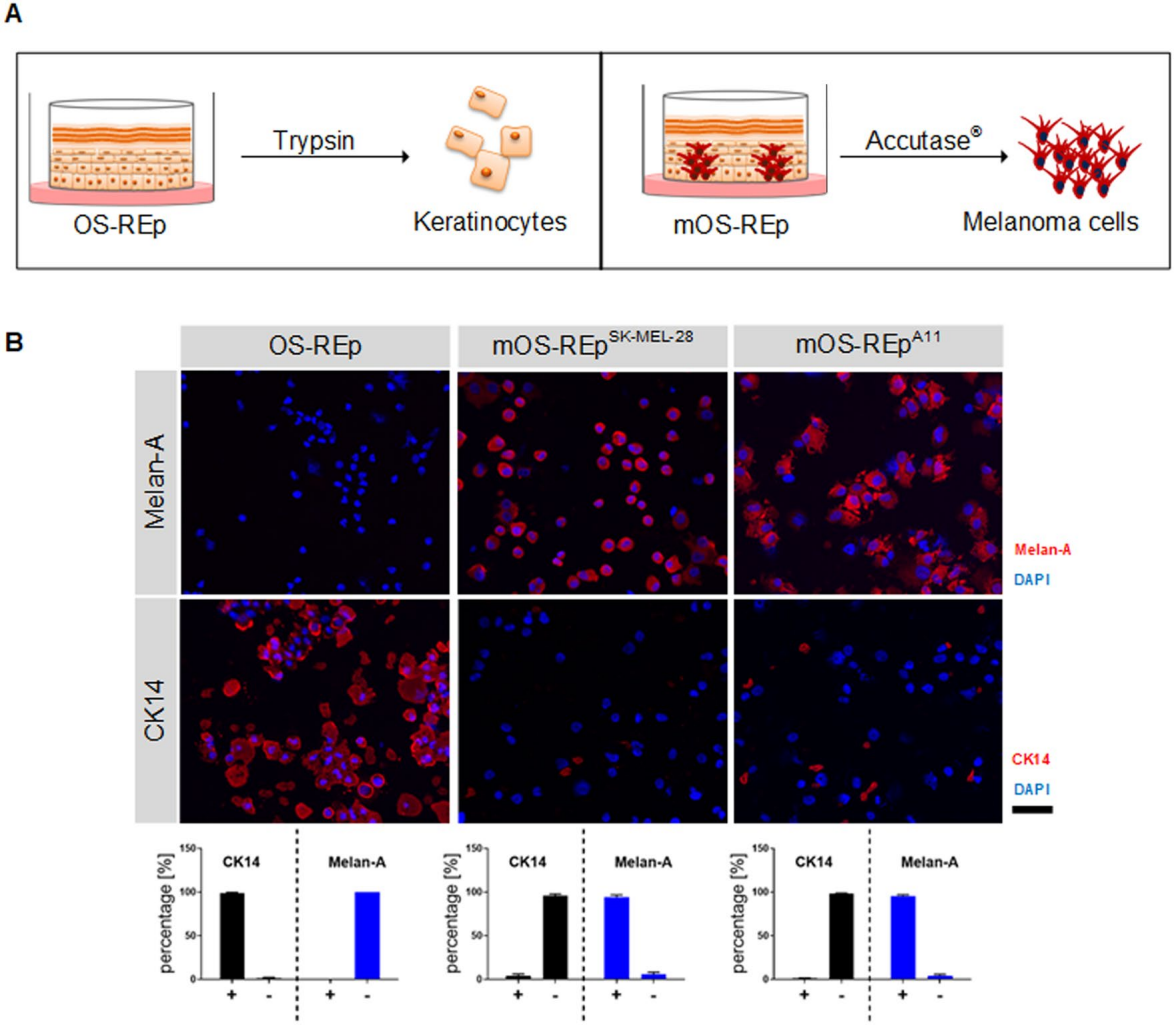
Re-Isolation of single cell suspensions via enzymatic digestion from melanoma and non-melanoma skin models. (**A**) Schematic of the re-Isolation process of single cells from the epidermal models. (**B**) Keratinocytes isolated via trypsin from OS-REp as well as melanoma cells isolated via accutase from mOS-REp were stained and quantified on cytospots with markers for keratinocytes (cytokeratin 14) and melanoma cells (Melan-A) (both in red). Cell nuclei were stained with DAPI (blue). Scale bar 50 µm.

To further investigate if our models capitulate in vivo conditions and for analysis of the impact of the 3D culture on the cells, we established a protocol to re-isolate single cells from our models. Single cells were segregated from the models by enzymatic digestion. Keratinocytes were isolated from OS-REp via trypsin, whereas melanoma cells were isolated from mOS-REp via accutase® (Fig. 3A). The enzyme as well as the incubation times were specifically chosen to allow re-isolation of melanoma cells from melanoma models and of keratinocytes from epidermal models. To test the effectiveness of this method, cytospots of the re-isolated cells were stained with markers for keratinocytes and melanoma cells. Cells re-isolated from OS-REp via trypsin were positive for the keratinocyte marker cytokeratin 14 and negative for the melanoma marker Melan-A (Fig. 3B). In contrast, cells re-isolated from mOS-REp via accutase® were positive for Melan-A and negative for cytokeratin 14. Red spots visible in the cytokeratin 14 staining of mOS-REp do not represent intact cells but only fragments that are formed during re-isolation processes.

**Figure 4:**
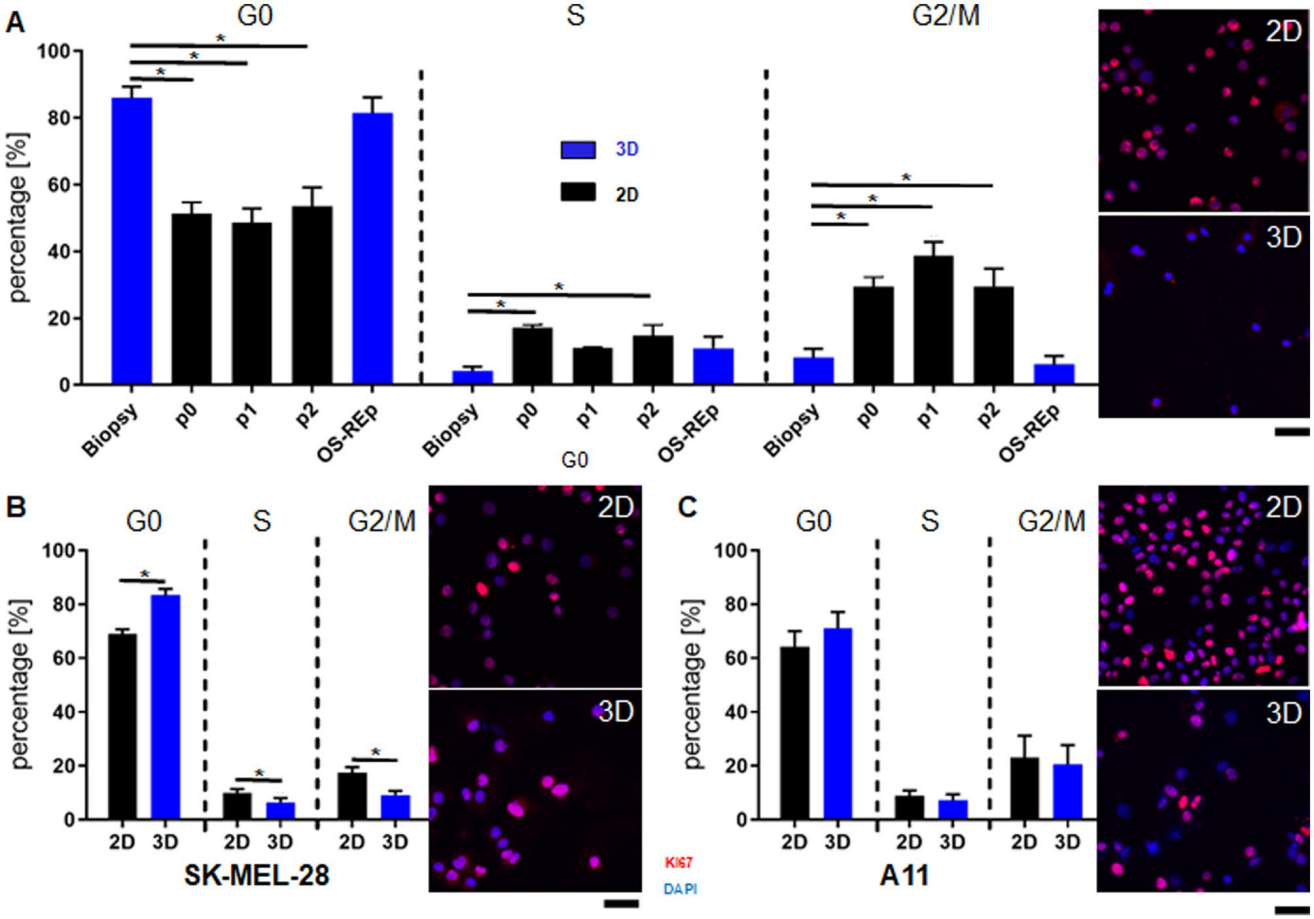
Analysis of 2D versus 3D culture. (**A**) Analysis of cell cycle and proliferation rates of keratinocytes revealed significant cell cycle differences of cells cultured in 2D (black bars) compared to cells cultured in 3D (blue bars). Immunofluorescence staining of the proliferation marker Ki67 (red) of cells on cytospots showed a higher amount of Ki67 positive cells in 2D than in cells re-isolated from 3D models. (**B**) Analysis of cell cycle and proliferation rates of the melanoma cell line SK-MEL-28. 3D culture led to an altered cell cycle and a slightly decreased Ki67 (red) staining in contrast to 2D. **(C**) Analysis of cell cycle and proliferation rates of the melanoma cell line A11 cultured in 2D compared to those cultured in 3D. Both, cell cycle and Ki67 (red) expression showed similar tendencies in 2D and 3D culture. Cell nuclei were stained with DAPI (blue). Scale bar 50 µm.

To check the influence of the 3D culture on the cells, we assessed the cell cycle and the proliferation of both, keratinocytes and melanoma cells re-isolated from our models, and compared these to cells in 2D culture. Cell cycle analysis revealed significant differences in the cell cycle of keratinocytes cultured in 2D compared to keratinocytes cultured in 3D (Fig. 4A). In all passages in 2D, there was a significant decrease of cells in G0/1 phase and an increase in S phase and G2/M phase compared to keratinocytes freshly isolated from skin biopsies. Keratinocytes re-isolated from OS-REp showed a similar cell cycle pattern to keratinocytes freshly isolated from human ex vivo epidermis. Histochemical analysis with Ki67 staining on cytospots allowed the identification of proliferative active cells. Keratinocytes in 2D showed a high amount of Ki67 positive cells. In comparison, cells re-isolated from OS-REp revealed a reduced Ki67 positive staining. Furthermore, for SK-MEL-28, 3D culture led to an altered cell cycle compared to 2D cell culture (Fig. 4B). The data showed a significant difference of cell cycle in all 3 phases and a slightly decreased Ki67 staining for 3D cultured cells. In contrast, the cell cycle of A11 showed a similar tendency but no significant difference and also the Ki67 staining was similar between the 2D and 3D cells (Fig. 4C).

### The BRAF inhibitor vemurafenib reduces tumor growth in mOS-REp^SK-MEL-28^ via cell cycle arrest

**Figure 5:**
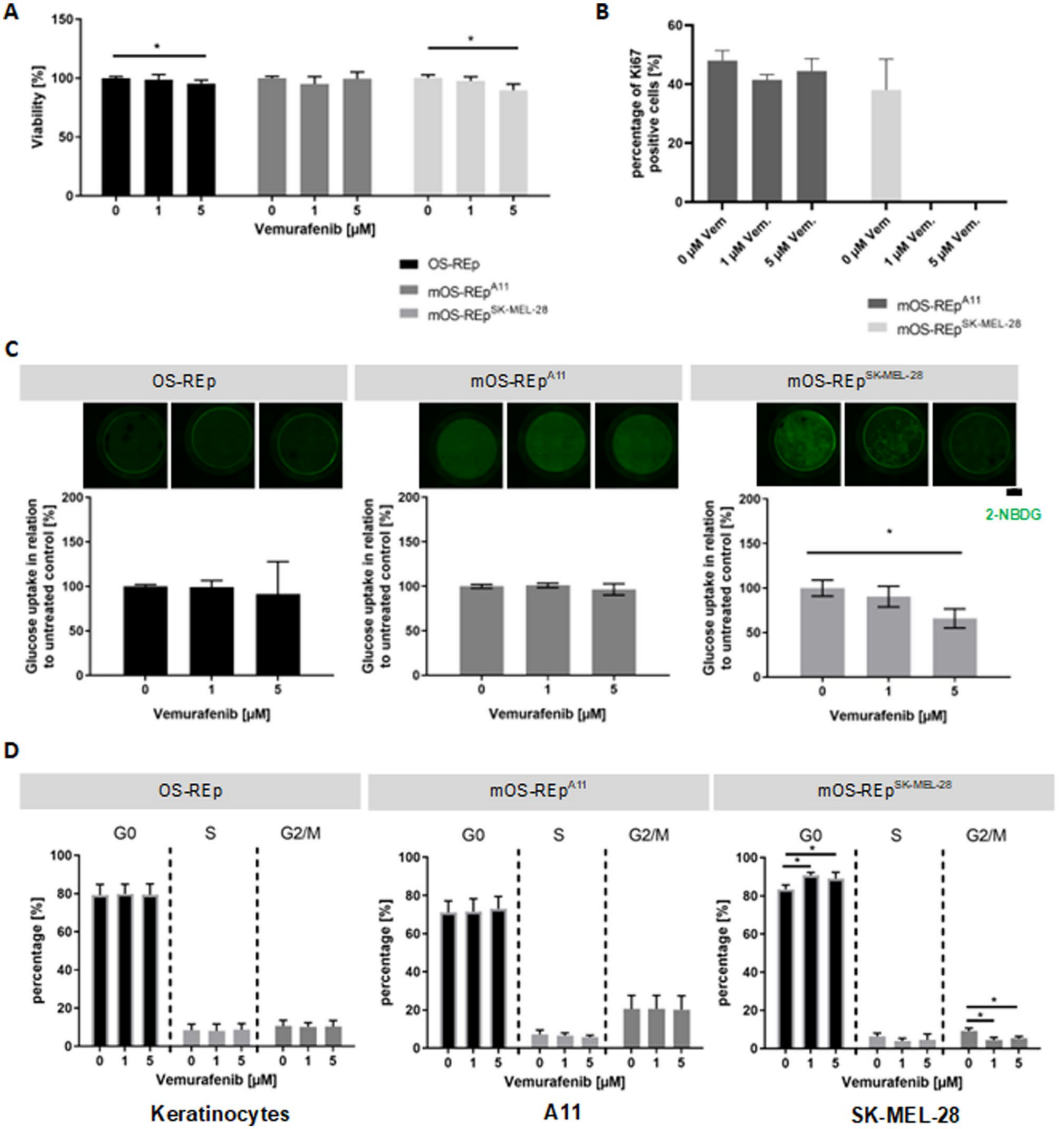
Qualification of mOS-REp as a test system for the assessment of tumor therapeutics. (**A**) Analysis of metabolic activity. mOS-REp^A11^ showed no significant difference in viability for different concentrations. In comparison, for OS-REp and mOS-REp^SK-MEL-28^ weak but statistically relevant decreases of viability were measurable for 5 µM vemurafenib. (**B**) Influence on proliferation rates. Melanoma cells re-isolated from BRAF^WT^ (mOS-REp^A11^) and BRAF^V600E^ (mOS-REp^SK-MEL-28^) melanoma skin equivalents were stained on cytospots (Fig. S2) with the proliferation marker Ki67 and counted quantitatively. In mOS-REp^SK-MEL-28^ the number of positively stained cells diminished completely after treatment, whereas the Ki67 expression was not altered by vemurafenib treatment in mOS-REp^A11^. (**C**) Measurement of glucose consumption. It was either directly measured in the supernatant or visualized locally by fluorescently labelled glucose. Both the fluorescent signal and glucose consumption decreased dose-dependently for mOS-REp^SK-MEL-28^. This could not be observed in mOS-REp^A11^. Scale bar 200 µm. (**D**) Treatment effects on the cell cycle. Cell cycle stages were determined by DNA content measured via flow cytometry after propidium iodide staining. Vemurafenib led to a dose-dependent significant increase of cells in G0 phase and decrease in G2/M phase in mOS-REp^SK-MEL-28^. OS-REp and mOS-REp^A11^ showed no treatment-dependent cell cycle response.

To investigate whether our established model is feasible as a test system for tumor therapeutics and whether in vivo results are reproducible, an established therapeutic was administered. Vemurafenib, a BRAF-inhibitor, clinically used in melanoma treatment^41,42^, was applied systemically to BRAF^WT^ (mOS-REp^A11^), BRAF^V600E^ (mOS-REp^SK-MEL-28^) and non-melanoma skin equivalents (Fig. 5). Since vemurafenib leads to an initial G0/G1 cell cycle arrest, followed by growth inhibition and consecutive cell death, we assessed the metabolic activity and glucose metabolism and analyzed differences in the cell cycle and proliferation rates after 72 h of treatment.

To analyze the metabolic activity of treated mOS-REp, a viability assay was performed directly in the model. For all experimental groups only a slight reduction of the viability was measured that reached statistical relevance for OS-REp and mOS-REp^SK-MEL-28^ at 5 µM (Fig. 5A).

Additionally, to investigate the influence of vemurafenib on proliferation rates, melanoma cells re-isolated from BRAF^WT^ (mOS-REp^A11^), BRAF^V600E^ (mOS-REp^SK-MEL-28^) and keratinocytes re-isolated from non-melanoma skin equivalents were stained with the proliferation marker Ki67 (Fig. 5B, Fig. S2). For OS-REp, Ki67 expression was generally low and not altered by vemurafenib treatment. In contrast, mOS-REp showed numerous Ki67-positive cells when untreated. While there was no significant response to the treatment in OS-REp and mOS-REp^A11^, the number of positively stained cells of mOS-REp^SK-MEL-28^ diminished completely after treatment with 1 µM and 5 µM vemurafenib.

Since melanoma cells show a high metabolic activity^43^, glucose consumption is associated with tumor progression. Thus, we hypothesized that treatment with vemurafenib will decrease glucose consumption which was either directly measured in the supernatant of cultured models or visualized by fluorescently labelled glucose (Fig. 5C). An increased fluorescence intensity and thus glucose uptake was shown for untreated mOS-REp^SK-MEL-28^ and mOS-REp^A11^ compared to OS-REp. Treatment with vemurafenib decreased the fluorescent signal dose-dependently for mOS-REp^SK-MEL-28^, which could not be observed in mOS-REp^A11^. Moreover, mOS-REp^SK-MEL-28^ showed 10% less glucose consumption after 1 µM and 35% after 5 µM of vemurafenib treatment.

Next, effects on the cell cycle of keratinocytes re-isolated from non-melanoma skin equivalents and melanoma cells re-isolated from both mOS-REp models were examined by flow cytometry (Fig. 5D). Vemurafenib treatment led to an altered cell cycle only in mOS-REp^SK-MEL-28^. While OS-REp and mOS-REp^A11^ showed no treatment-dependent cell cycle response (on average for OS-REp 79.5% ± 5.4% of cells were detectable in G0 phase and 10.4% ± 2.7% of cells in G2/M phase, for mOS-REp^A11^ 71.9% ± 6.4% of cells were detectable in G0 phase and 20.5% ± 7.1% of cells in G2/M phase), vemurafenib led to a dose-dependent significant increase of cells in G0 phase (from 83.4% ± 2.4% at 0 µM to 90.7% ± 1.5% at 1 µM and 89.0% ± 3.3% at 5 µM) and decrease in G2/M phase in mOS-REp^SK-MEL-28^ (from 9.2% ± 1.5% at 0 µM to 4.5% ± 1.3% at 1 µM and 5.3% ± 1.0% at 5 µM).

## Discussion

The worldwide incidence of melanoma has increased tremendously over the last five decades^44^, emphasizing the need for further research tools and predictive models. For this aim, we established a model mimicking melanoma within the epidermis.

Other models introduced as preclinical test platforms for in vitro drug discovery are ranging from di-, tri- or multi-cellular spheroid melanoma^45^, but often lack an appropriate reflection of the tissue, in which the tumor develops. Therefore, more complex models have been developed such as co-culture models with skin cells^46^, a sheet-based melanoma model^23^ and a human skin equivalent on-a-chip platform^47^. However, the standardization and thus the industrial applicability of these models for preclinical drug testing is limited. The increased complexity of these test systems is detrimental to reproducibility and scale up. Organotypic 3D models should aim to recreate the cellular microenvironment with its crucial cell–cell and cell–matrix interactions as simple as possible^6^. Thus, less complex 3D models with a limited number of variables mimicking the melanoma microenvironment are needed. In the normal epidermis, keratinocytes regulate proliferation and localization of melanocytes. During the transformation of melanocytes to melanoma cells, tumor cells escape the tight control of keratinocytes by downregulation of adhesion molecules^48,49^. Hence, we focused on the key microenvironment of melanoma development: the epidermis. Thus, in contrast to the widely used epidermal-dermal melanoma models^15–26^, the here described model comprises only the epidermis. While one could argue that by omitting the dermis a large part of the organotypic microenvironment is missing and hence metastasis or the influence of dermal fibroblasts cannot be investigated, this model has some advantages: The RHE is standardizable and therefore already accepted in internationally approved test guidelines^50,51^. The culture time is shorter (approximately 3 weeks for epidermal models compared to 4–6 weeks for epidermal-dermal models) and the amount of cell culture medium is reduced, thereby lowering the costs. Fewer cell types facilitate handling, enabling high-throughput and up-scaling.

Since different melanomas vary regarding aggressiveness, origin and driver mutations, we systematically assessed ten cell lines with different driver mutations using the same experimental approach (Table 1, Fig. S1). The morphology of the mOS-REp was comparable to that present in vivo as the models show the development of all epidermal layers with melanoma clusters growing in the basal layer. For mOS-REp^A11^ and mOS-REp^SK-MEL-28^ immunohistological analysis revealed expression of common melanoma markers (Fig. 1C) comparable to skin melanoma in vivo. Although the epidermal morphology was significantly interrupted by the tumor nests, the overall barrier function of the models was not impaired (Fig. 1D), attributable to the presence of an intact *stratum corneum,* the main contributor to the skin barrier. However, employing the melanoma cell lines MM127, D08, A375, Malme3M and BLM, mOS-REp generation was not successful since the surrounding keratinocytes might suppress tumor progression and thus reduce the cellular growth in the models. However, it should be noted that the visualization of the BLM and MM127 cell line is challenging due to a loss of standard melanoma marker^52–54^.

Melanomagenesis is associated with a significant change in the metabolic activity of tumor cells, which is expressed by an increased glucose turnover rate followed by intensified lactate production. This enhanced glycolytic metabolization of glucose in presence of oxygen was originally observed by Otto Warburg^55,56^. At the same time, the tumor cells change the metabolic composition of the surrounding extracellular milieu and thus influence the tumor microenvironment. Comparable to in vivo, the overall glucose consumption of the melanoma models was increased for both cell lines. Moreover, a locally increased glucose consumption within the melanoma nests could be detected by using fluorescence-labelled glucose (Fig. 1E) whereas 2-P FLIM measured non-invasively the metabolic shift occurring between melanoma and healthy cells. In both melanoma models the keratinocytes have statistically significant longer fluorescence lifetime, which translates to a higher dependence of OxPhos whilst the melanoma has shorter fluorescence lifetimes reflective of increased dependence on glycolysis (Fig. 2C). This occurs in order to adapt and facilitate the uptake and incorporation of nutrients for biosynthesis needed for proliferation^33,57–59^.

To further probe cellular metabolism using 2-P FLIM, FAD^+^ fluorescence intensity was also acquired, however, no statistical significance was verified when comparing keratinocytes with melanoma cells (Fig. 2D). An important metabolic characteristic of tumor cells associated with higher glucose consumption and lactate secretion is the higher consumption of glutamine^60–63^. Increased levels of glutamine consumption promote a decrease in the ORR values as observed by Varone et al.^64^. ORR values are higher than expected in melanoma cells and have similar values as the keratinocytes (Fig. 2D). This can be a direct result of reverse flow of the TCA cycle by reductive carboxylation of α-ketoglutarate observed in previous studies^65,66^. This reaction step can consume the excess of NADH being produced during the conversion of glutamate to α-ketoglutarate^67,68^.

Since melanoma skin models are generated from keratinocytes and melanoma cells, the analysis of such models requires specific methods that allow for a deconvolution of the effects on both cell populations. We specifically re-isolated either keratinocytes or melanoma cells (Fig. 3A) by utilizing the altered cell-to-cell adhesion^69^. Using this method, we were able to employ flow cytometry to analyze the proliferation rate of the two cell types separately. Under 2D culture conditions more cells undergo mitosis than in the 3D model. Although the trend is observable for both cell lines, the effect reaches only statistical significance for SK-MEL-28. A reduced proliferation for tumor cell lines in 3D has been shown in numerous studies and was found to reflect the situation in native tumor tissue more correctly^66^. Preclinical assessment of novel drugs requires a comparable proliferation to accurately predict the outcome in a human situation. Hence, the reduced proliferation was found to be a key advantage of 3D over simple 2D cell models that are prone to overpredict the effect of a novel therapy^70^.

Although being suitable for early stages of drug development (Fig. 5), one should bear in mind that the here presented mOS-REp is lacking some cellular components to mimic the complete tumor environment. The approach balances between the above stated advantages of having a solely epidermal model and the risk of leaving out important features by reducing the model to the epidermis. The epidermal model can only reflect melanoma-keratinocyte interactions, whereas other cell influences are not included. However, the choice of the model should be dependent on the kind of scientific question and the here presented model enhances the available options. To study further tumor progression, other structures such as the vascular or lymphatic system could be included in a more refined model with low throughput but high in vivo correlation. By integrating T cells, immuno-competent models could serve as a test platform to evaluate the efficacy of immunotherapeutics such as checkpoint inhibitors. Due to patient specific differences and the cellular heterogeneity, only a small subset of patients responds to standard antitumor therapies. Thus, personalized medicine will be the next step in cancer therapies and test systems need to address these population differences to mimic the response in clinical cohorts. Employing patient-derived tumor cells to generate mOS-REp, the model could be used as a novel tool in personalized medicine e.g. for the prediction of individual therapies.

## Supplementary Information

Supplementary Figures.

## Data Availability

The datasets generated during the current study are available from the corresponding author on reasonable request.

## References

[1] Miller AJ, Mihm MC, Melanoma JR (2006). Microorganisms in the phyllosphere of temperate forest ecosystems in a changing. N. Engl. J. Med..

[2] Garbe C (2010). Diagnosis and treatment of melanoma: European consensus-based interdisciplinary guideline. Eur. J. Cancer.

[3] Damsky WE, Rosenbaum LE, Bosenberg M (2010). Decoding melanoma metastasis. Cancers.

[4] Winder M, Virós A (2018). Mechanisms of drug resistance in melanoma. Handb. Exp. Pharmacol..

[5] Rastrelli M, Tropea S, Rossi CR, Alaibac M (2014). Melanoma: Epidemiology, risk factors, pathogenesis, diagnosis and classification. In Vivo.

[6] Smalley KSM, Lioni M, Noma K, Haass NK, Herlyn M (2008). In vitro three-dimensional tumor microenvironment models for anticancer drug discovery. Expert Opin. Drug Discov..

[7] Beaumont KA, Mohana-Kumaran N, Haass NK (2013). Modeling melanoma in vitro and in vivo. Healthcare.

[8] Hoffmann TK (2009). A novel mechanism for anti-EGFR antibody action involves chemokine-mediated leukocyte infiltration. Int. J. Cancer.

[9] Lazzari G (2018). Multicellular spheroid based on a triple co-culture: A novel 3D model to mimic pancreatic tumor complexity. Acta Biomater..

[10] Vinci M (2012). Advances in establishment and analysis of three-dimensional tumor spheroid-based functional assays for target validation and drug evaluation. BMC Biol..

[11] Zanoni M (2016). 3D tumor spheroid models for in vitro therapeutic screening: a systematic approach to enhance the biological relevance of data obtained. Sci. Rep..

[12] Marconi A, Quadri M, Saltari A, Pincelli C (2018). Progress in melanoma modelling in vitro. Exp. Dermatol..

[13] Mueller-Klieser W (1987). Multicellular spheroids: A review on cellular aggregates in cancer research. J. Cancer Res. Clin. Oncol..

[14] Lin R-Z, Chang H-Y (2008). Recent advances in three-dimensional multicellular spheroid culture for biomedical research. Biotechnol. J..

[15] Meier F (2000). Human melanoma progression in skin reconstructs: biological significance of bFGF. Am. J. Pathol..

[16] Eves P (2000). Characterization of an in vitro model of human melanoma invasion based on reconstructed human skin. Br. J. Dermatol..

[17] Li L, Fukunaga-Kalabis M, Herlyn M (2011). The three-dimensional human skin reconstruct model: A tool to study normal skin and melanoma progression. J. Vis. Exp. JoVE..

[18] Gibot L, Galbraith T, Huot J, Auger FA (2013). Development of a tridimensional microvascularized human skin substitute to study melanoma biology. Clin. Exp. Metast..

[19] Vörsmann H (2013). Development of a human three-dimensional organotypic skin-melanoma spheroid model for in vitro drug testing. Cell Death Dis..

[20] Hill DS (2015). A novel fully humanized 3D skin equivalent to model early melanoma invasion. Mol. Cancer Ther..

[21] Marques CMG, MacNeil S (2016). Use of a tissue engineered human skin model to investigate the effects of wounding and of an anti-inflammatory on melanoma cell invasion. PLoS ONE.

[22] Haridas P, McGovern JA, McElwain SDL, Simpson MJ (2017). Quantitative comparison of the spreading and invasion of radial growth phase and metastatic melanoma cells in a three-dimensional human skin equivalent model. PeerJ.

[23] Bourland J, Fradette J, Auger FA (2018). Tissue-engineered 3D melanoma model with blood and lymphatic capillaries for drug development. Sci. Rep..

[24] Commandeur S (2014). In-vitro melanoma models: invasive growth is determined by dermal matrix and basement membrane. Melan. Res..

[25] Michielon E (2020). Micro-environmental cross-talk in an organotypic human melanoma-in-skin model directs M2-like monocyte differentiation via IL-10. Cancer Immunol. Immunother..

[26] Patton EE (2021). Melanoma models for the next generation of therapies. Cancer Cell.

[27] Global Data. *Global Data-Drug Data Base*. https://pharma.globaldata.com (2020).

[28] Russell WMS, Burch RL (1960). The principles of humane experimental technique. Med. J. Austral..

[29] Groeber F (2016). Catch-up validation study of an in vitro skin irritation test method based on an open source reconstructed epidermis (phase II). Toxicol. In Vitro.

[30] Alexander H, Brown S, Danby S, Flohr C (2018). research techniques made simple: Transepidermal water loss measurement as a research tool. J. Investig. Dermatol..

[31] Kiesewetter L, Littau L, Walles H, Boccaccini AR, Groeber-Becker F (2019). Reepithelialization in focus: Non-invasive monitoring of epidermal wound healing in vitro. Biosens. Bioelectrons.

[32] Van Heiden MG, Cantley LC, Thompson CB (2009). Understanding the Warburg effect: The metabolic requirements of cell proliferation. Science.

[33] Hsu PP, Sabatini DM (2008). Cancer cell metabolism: Warburg and beyond. Cell.

[34] Okkelman IA, Neto N, Papkovsky DB, Monaghan MG, Dmitriev RI (2020). A deeper understanding of intestinal organoid metabolism revealed by combining fluorescence lifetime imaging microscopy (FLIM) and extracellular flux analyses. Redox Biol..

[35] Floudas A (2020). Pathogenic, glycolytic PD-1+ B cells accumulate in the hypoxic RA joint. JCI Insight.

[36] Skala MC (2007). In vivo multiphoton microscopy of NADH and FAD redox states, fluorescence lifetimes, and cellular morphology in precancerous epithelia. Proc. Natl. Acad. Sci. USA.

[37] Meleshina AV (2017). Two-photon FLIM of NAD(P)H and FAD in mesenchymal stem cells undergoing either osteogenic or chondrogenic differentiation. Stem Cell Res. Ther..

[38] Walsh AJ (2021). Classification of T-cell activation via autofluorescence lifetime imaging. Nat. Biomed. Eng..

[39] Ostrander JH (2010). Optical redox ratio differentiates breast cancer cell lines based on estrogen receptor status. Cancer Res..

[40] Alhallak K, Rebello LG, Muldoon TJ, Quinn KP, Rajaram N (2016). Optical redox ratio identifies metastatic potential-dependent changes in breast cancer cell metabolism. Biomed. Opt. Express.

[41] Bollag G (2012). Vemurafenib: The first drug approved for BRAF-mutant cancer. Nat. Rev. Drug Discov..

[42] Chapman PB (2011). Improved survival with vemurafenib in melanoma with BRAF V600E mutation. N. Engl. J. Med..

[43] Ratnikov BI, Scott DA, Osterman AL, Smith JW, Ronai ZA (2017). Metabolic rewiring in melanoma. Oncogene.

[44] Rigel DS, Carucci JA (2000). Malignant melanoma: Prevention, early detection, and treatment in the 21st century. CA Cancer J. Clin..

[45] Klicks J, Maßlo C, Kluth A, Rudolf R, Hafner M (2019). A novel spheroid-based co-culture model mimics loss of keratinocyte differentiation, melanoma cell invasion, and drug-induced selection of ABCB5-expressing cells. BMC Cancer.

[46] Morales D (2019). 3D coculture models underline metastatic melanoma cell sensitivity to vemurafenib. Tissue Eng. A.

[47] Abaci HE, Gledhill K, Guo Z, Christiano AM, Shuler ML (2015). Pumpless microfluidic platform for drug testing on human skin equivalents. Lab Chip.

[48] Gurzu S, Beleaua MA, Jung I (2018). The role of tumor microenvironment in development and progression of malignant melanomas: A systematic review. Roman. J. Morphol. Embryol..

[49] Villanueva J, Herlyn M (2008). Melanoma and the tumor microenvironment. Curr. Oncol. Rep..

[50] OECD. *Test No. 431: In Vitro Skin Corrosion: Reconstructed Human Epidermis (RHE) Test Method. OECD Guidelines for the Testing of Chemicals, Section 4* (Paris, 2019).

[51] OECD. *Test No. 439: In Vitro Skin Irritation: Reconstructed Human Epidermis Test Method. OECD Guidelines for the Testing of Chemicals, Section 4* (Paris, 2021).

[52] Kobayashi J, Fujimoto D, Murakami M, Hirono Y, Goi T (2018). A report of amelanotic malignant melanoma of the esophagus diagnosed appropriately with novel markers: A case report. Oncol. Lett..

[53] Oiso N, Yoshida M, Kawara S, Kawada A (2010). Amelanotic vulvar melanoma with intratumor histological heterogeneity. J. Dermatol..

[54] Haridas P, McGovern JA, Kashyap AS, McElwain DLS, Simpson MJ (2016). Standard melanoma-associated markers do not identify the MM127 metastatic melanoma cell line. Sci. Rep..

[55] Warburg O (1924). Über den stoffwechsel der carcinomzelle. Naturwissenschaften.

[56] Warburg O (1956). On the origin of cancer cells. Science.

[57] DeBerardinis RJ, Lum JJ, Hatzivassiliou G, Thompson CB (2008). The biology of cancer: Metabolic reprogramming fuels cell growth and proliferation. Cell Metab..

[58] Provenzano PP, Eliceiri KW, Keely PJ (2009). Multiphoton microscopy and fluorescence lifetime imaging microscopy (FLIM) to monitor metastasis and the tumor microenvironment. Clin. Exp. Metast..

[59] Walsh A, Cook RS, Rexer B, Arteaga CL, Skala MC (2012). Optical imaging of metabolism in HER2 overexpressing breast cancer cells. Biomed. Optics Express.

[60] Yang L, Venneti S, Nagrath D (2017). Glutaminolysis: A hallmark of cancer metabolism. Annu. Rev. Biomed. Eng..

[61] Dang CV (2010). Glutaminolysis: Supplying carbon or nitrogen or both for cancer cells?. Cell Cycle.

[62] DeBerardinis RJ, Cheng T (2010). Q's next: The diverse functions of glutamine in metabolism, cell biology and cancer. Oncogene.

[63] Pérez-Escuredo J (2016). Lactate promotes glutamine uptake and metabolism in oxidative cancer cells. Cell Cycle.

[64] Varone A (2014). Endogenous two-photon fluorescence imaging elucidates metabolic changes related to enhanced glycolysis and glutamine consumption in precancerous epithelial tissues. Cancer Res..

[65] Scott DA (2011). Comparative metabolic flux profiling of melanoma cell lines: Beyond the Warburg effect. J. Biol. Chem..

[66] Fischer GM (2018). Metabolic strategies of melanoma cells: Mechanisms, interactions with the tumor microenvironment, and therapeutic implications. Pigment Cell. Melanoma Res..

[67] Rodrigues MF (2016). Enhanced OXPHOS, glutaminolysis and beta-oxidation constitute the metastatic phenotype of melanoma cells. Biochem. J..

[68] Filipp FV, Scott DA, Ronai ZA, Osterman AL, Smith JW (2012). Reverse TCA cycle flux through isocitrate dehydrogenases 1 and 2 is required for lipogenesis in hypoxic melanoma cells. Pigment. Cell Melanoma Res..

[69] Haass NK, Smalley KSM, Herlyn M (2004). The role of altered cell-cell communication in melanoma progression. J. Mol. Histol..

[70] Edmondson R, Broglie JJ, Adcock AF, Yang L (2014). Three-dimensional cell culture systems and their applications in drug discovery and cell-based biosensors. Assay Drug Dev. Technol..

