# Supplementary material for: A preclinical model of cutaneous melanoma based on reconstructed human epidermis
Source: Sci Rep. 2022 Sep 29;12:16269. doi: 10.1038/s41598-022-19307-0 (PMC9522649; doi:10.1038/s41598-022-19307-0)
Supplement: Supplementary file 1 — Supplementary Figures. [file 41598_2022_19307_MOESM1_ESM.docx]

# Supplements


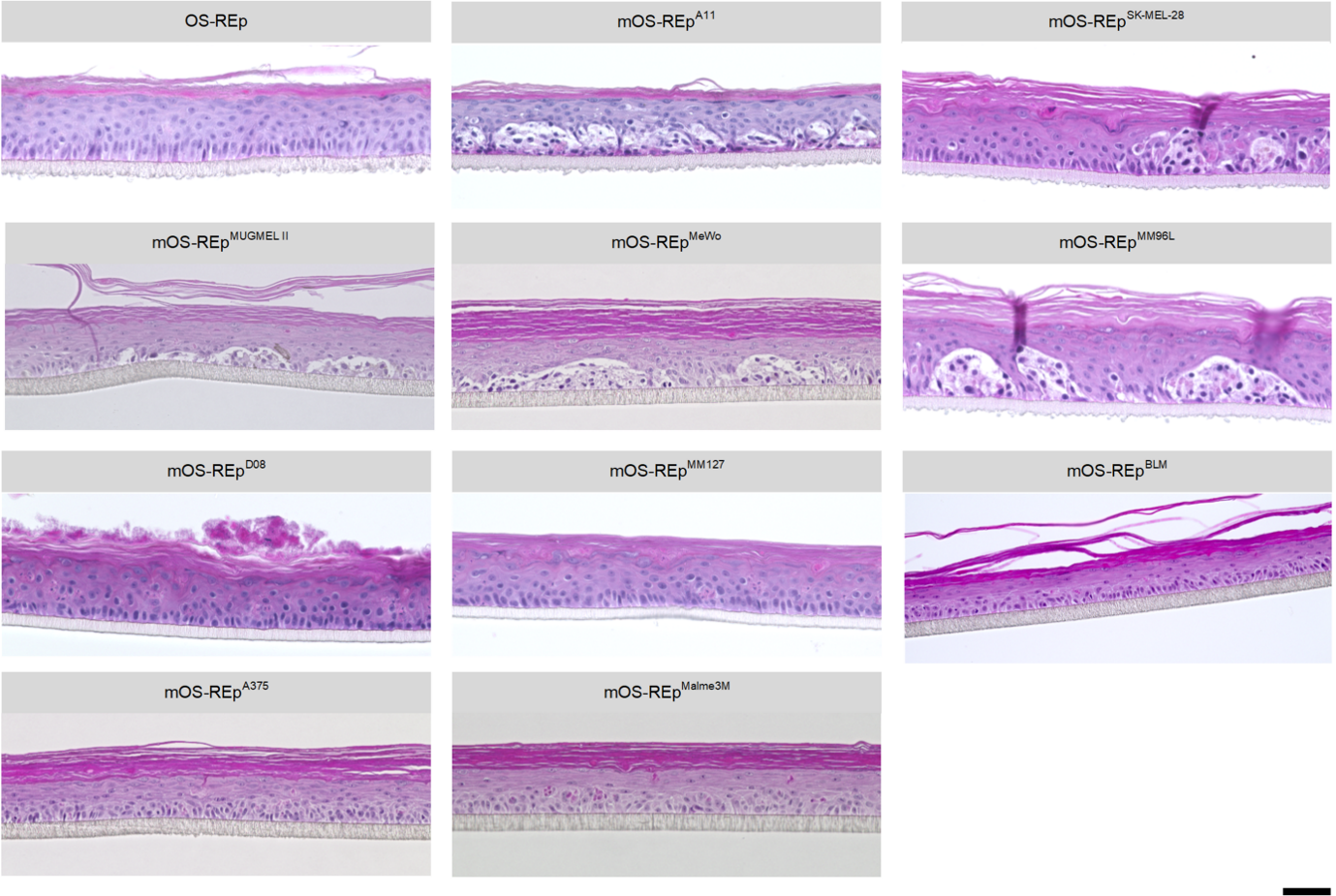


**Supplementary Figure 1: Incorporation of different melanoma cell lines in OS-Rep.** Hematoxylin and eosin staining of cross-sections of mOS-REp with different melanoma cell lines. mOS-REp were morphologically comparable to OS-REp, as they showed a multilayer epidermis, consisting of *stratum basale*, *stratum spinosum, stratum granulosum and stratum corneum.* Micro tumors formed sucessfully in mOS-REp^A11^, mOS-REp^SK-MEL-28^, mOS-REp^MUGMELII^, mOS-REp^MeWo^ and mOS-REp^MM96L^ appeared as local spots of loose tissue. Scale bar 50 µm.


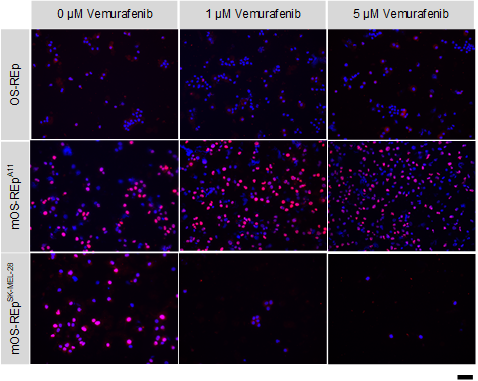


**Supplementary Figure 2:** **Influence of vemurafenib treatment on proliferation rates**. Melanoma cells re-isolated from BRAF^WT^ (mOS-REp^A11^) and BRAF^V600E^ (mOS-REp^SK-MEL-28^) melanoma skin equivalents and keratinocytes re-isolated from non-melanoma skin equivalents were stained on cytospots with the proliferation marker Ki67. In mOS-REp^SK-MEL-28^ the number of positively stained cells diminished completely after treatment, whereas the Ki67 expression was not altered by Vemurafenib treatment in OS-REp and mOS-REp^A11^. Cell nuclei were stained with DAPI. Scale bar 50 µm.
